# Marine Actinomycetes Associated with Stony Corals: A Potential Hotspot for Specialized Metabolites

**DOI:** 10.3390/microorganisms10071349

**Published:** 2022-07-04

**Authors:** Galana Siro, Atanas Pipite, Ketan Christi, Sathiyaraj Srinivasan, Ramesh Subramani

**Affiliations:** 1 School of Agriculture, Geography, Environment, Ocean and Natural Sciences (SAGEONS), The University of the South Pacific, Laucala Campus, Suva, Fiji; sirogalana@yahoo.com (G.S.); ketan.christi@usp.ac.fj (K.C.); ramesh.subramani@usp.ac.fj (R.S.); 2 Department of Bio & Environmental Technology, Division of Environmental & Life Science, College of Natural Science, Seoul Women’s University, 623 Hwarangno, Nowon-gu, Seoul 01797, Korea

**Keywords:** marine actinomycetes, actinobacteria, microbial secondary metabolite, scleractinian coral, antimicrobial resistance

## Abstract

Microbial secondary metabolites are an important source of antibiotics currently available for combating drug-resistant pathogens. These important secondary metabolites are produced by various microorganisms, including Actinobacteria. Actinobacteria have a colossal genome with a wide array of genes that code for several bioactive metabolites and enzymes. Numerous studies have reported the isolation and screening of millions of strains of actinomycetes from various habitats for specialized metabolites worldwide. Looking at the extent of the importance of actinomycetes in various fields, corals are highlighted as a potential hotspot for untapped secondary metabolites and new bioactive metabolites. Unfortunately, knowledge about the diversity, distribution and biochemistry of marine actinomycetes compared to hard corals is limited. In this review, we aim to summarize the recent knowledge on the isolation, diversity, distribution and discovery of natural compounds from marine actinomycetes associated with hard corals. A total of 11 new species of actinomycetes, representing nine different families of actinomycetes, were recovered from hard corals during the period from 2007 to 2022. In addition, this study examined a total of 13 new compounds produced by five genera of actinomycetes reported from 2017 to 2022 with antibacterial, antifungal and cytotoxic activities. Coral-derived actinomycetes have different mechanisms of action against their competitors.

## 1. Introduction

More than 30,000 diseases have been clinically documented due to their severity that puts people’s health and livelihoods at risk [1]. In addition, multidrug-resistant (MDR) human pathogens or “superbugs” continue to pose a serious threat to global health due to their emergence, widespread transmissibility and persistence [2]. However, despite the tremendous advances in technology, science and medicine during this era, there is a huge difficulty in preventing the proliferation of these superbugs [3]. Globally, 700,000 people die each year from resistant infections, and it is estimated that by 2050, antimicrobial resistance (AMR) could claim the lives of 10 million people each year without intervention [4]. The estimated death toll is quite alarming as the world ventures into an unpredictable future. According to the recent publication of the World Health Organization (WHO), it classifies MDR pathogens into different priorities, ranging from priority 1 to priority 3 [5]. Although drug resistance is a natural occurrence, the indiscriminate misuse, overuse and underuse of pharmaceutical agents such as antibiotics continue to allow MDR microbes to adapt by developing sophisticated defense mechanisms. The latter may be by the pumping of antibiotics via a transmembrane efflux pump, chromosomal mutation and the production of antibiotic digestive enzymes [6]. Antibiotics are occasionally released into the environment through various routes and are stored in the soil and groundwater, which are the main reservoirs of antibiotic residues [7]. The persistent problem of the poor quality and poor administration of antimicrobial drugs has stimulated pathogens to develop resistance effectively and efficiently within days. As a counter-approach, the development and treatment of antibiotics is one of the main approaches of modern medicine in the fight against new diseases and antimicrobial resistance [2]. However, the multidrug resistance crisis is increasing dramatically. It has been reported that more than 70% of pathogenic bacteria are resistant to currently available antibiotics [8]. This obviously proves that the rampage of MDR pathogens is increasing in scale and is probably more catastrophic than in previous years. Since microbes and their resistant genes are able to adapt to changes in their environment [8], there is an urgent need to produce new antibiotics to fight against progressive bacterial infections in the world.

The need to acquire profitable molecules continues to prompt scientists to focus on unusual habitats with the prospect of isolating new natural products to meet growing pharmaceutical demand and combat MDR pathogens. An unusual habitat represented in this review is ocean biodiversity, which includes marine habitats and their microbiomes. This review focuses specifically on the phylum Actinobacteria or Actinomycetota, a taxon that covers a range of gifted bacterial clades known to synthesize the majority of available antibiotics approved for use [9]. We review, in particular, data from marine actinomycetes associated with hard corals, especially in the order Scleractinia. Marine actinomycetes recovered from hard corals are known to produce specialized metabolites of pharmaceutical interest. This review presents practical ideas and encouraging results to help researchers meet the challenges that are preventing progress to find novel antibiotics from marine actinomycetes associated with hard corals. Recent studies on new species and compounds of actinobacteria discovered from hard corals from 2007 to 2022 are summarized in this review. 

## 2. The Ocean Habitat and Microbial Diversity: A New Leaf in Drug Discovery

Oceans are the largest and most dynamic ecosystems covering 71% of the Earth’s surface, and which are represented by 32 of the 34 animal phyla, 15 of which are exclusively marine [10]. Their usual and unusual ecosystems play a major role in the biology of this planet in terms of the origin and evolution of life [11]. Moreover, their extensive dominance persists as a rich reservoir of microorganisms, explored or elusive [12]. Of all the taxonomically characterized species of organisms, microorganisms are undoubtedly the most diverse [13]. It is documented that 5 × 10^30^ of microbial life inhabit the earth [14]. Microbes are dispersed throughout all of the earth’s habitats with inherent physiological and functional diversity [15]. They are sensitive to changes in environmental parameters because they acquire a large surface/volume ratio due to their microscopic size [16]. Changes in physicochemical factors can influence or alter the functional dynamics of a microbiome [17]. Competition for survival along with adverse environmental stress are phenomena that have driven microbes’ evolution of defense, attack and signaling mechanisms, resulting in biological and chemical diversity [18]. The biogeography of microorganisms offers a huge resource of intact and effective therapeutic metabolites [19]. Considering the vast surface of the oceans, their microbiological environments are very complex with microbial abundances of 10^6^ per ml of seawater and 10^9^ per ml in the bottom of oceanic sediments [20], and an overall estimate of the microbial composition of 3.5 × 10^30^ [14,21]. Marine microorganisms live in abundance as plankton in water columns, biofilm in benthic substrates and symbionts in marine organisms [21,22]. The vast body of knowledge about marine microbial diversity is determined by conventional culture-independent and -dependent methods [23]. Since microorganisms are an integral part of the ocean, which comprises 90% of the ocean’s biomass, they play a very crucial role in biogeochemical cycles and biological processes [21].

Studies have shown that the number of bioactive agents of microbial origin in the terrestrial environment has declined steadily since the late 1980s, while the rate of rediscovery of known compounds has increased [24]. This problem arises mainly because of the redundancy of the screening techniques that include the type of technology, as well as the samples and the isolated strains of interest [25]. In this regard, alternative measures or strategies are being explored to overcome this stagnation in drug discovery. Therefore, many studies have made bioprospecting in marine habitats a new frontier of research due to its diverse metabolic capacities produced by a wide range of microorganisms [26,27]. The vast limits of the ocean are presented as an unexplored area of opportunity. Since the 1960s, the first research on microbial products from the oceans has been difficult and limited to habitats such as the intertidal and subtidal regions [28]. With the recent introduction of scuba diving and, later, scuba diving boats, the scope for screening for bioactive metabolites in deeper waters has increased [29]. The marine environment includes several types of habitats with unusual parameters such as high salinity, high pressure, fluctuating temperatures, and low or no light intensity contributing to a wide range of biodiversity, which varies greatly with depth [30]. Despite this, microbes must adapt to both the presence of competitive species and changing environmental conditions to increase their fitness. This involves adaptation via physiological mechanisms and the biochemical production of unique natural products [31,32]. There is a great disparity between the marine environment and the terrestrial environment in terms of biological and chemical diversity [33]. This great variability coordinated the speciation of oceanic biodiversity at all phylogenetic levels [22]. Therefore, due to these oceanic peculiarities, natural marine products are more robust and bioactive than those of their terrestrial counterparts, thus having a higher chance of drug discovery [34]. The ocean, being the greatest biodiversity on earth, represents a treasure trove of new beneficial natural products [35,36]. These ranges of natural products can be obtained from invertebrates and marine microorganisms. Natural products, mainly secondary metabolites exuded in the ocean, are rapidly diluted, so these compounds are programmed to have greater potent activity in order to be effective in the water column [37]. These ranges of natural products can be obtained from invertebrates and marine actinomycetes.

## 3. Actinomycetes: A Teeming Wealth of Chemical and Biological Warfare

The phylum Actinomycetota (Actinobacteria) is one of the main branches or lineages of bacteria (http://www.bacterio.net/-classifphyla.html#actinobacteria, accessed on 1 September 2021).

This group of bacteria has different mechanisms of action, which make them a major natural source of antibiotics. Hereinafter, the term actinomycetes refers to members of the phylum Actinomycetota (Actinobacteria). Actinobacteria, sometimes called actinomycetes, are Gram-positive bacteria that can be unicellular (e.g., *Arthrobacter* spp.) or multicellular (e.g., *Streptomyces* spp.) with non-spore- and spore-forming abilities. They contain sufficient guanine and cytosine contents in their genome and are predominantly aerobic with a few anaerobic and facultative exceptions, but are phylogenetically diverse exhibiting filamentous characteristics [38]. Although actinomycetes share a morphological resemblance to both fungi and other bacteria, their high GC content separates them as a distinct bacterial group. Actinomycetes, including both symbionts (e.g., *Frankia* spp.) and pathogens (e.g., Corynebacterium diphtheriae) are free-living, saprophytic and ubiquitous in terrestrial and aquatic environments. They are the most biotechnological and economical microorganisms that live under the most diverse conditions [39]. In addition, actinomycete is one of the major contributors to the production of geosmin and 2-methylisoborneol (2-MIB). These secondary compounds are tertiary alcohols, widely known to cause earthy and musty odors [40]. Actinomycetes appear to be very competitive under adverse environmental conditions due to certain properties they possess. First, they can produce a wide range of spores in a very short period of vegetative growth. Second, actinomycetes are versatile in terms of acquiring nutrition. Their growth is viable whether there is the sufficient availability of the minimum amount of nutrients or no nutrients. Third, having the ability to grow mycelium coordinates the colonization of nutrients away from the growth center. Finally, actinomycetes are able to synthesize compounds that prevent them from undergoing microbial degradation as well as the production of secondary metabolites, which are used to their advantage against other microorganisms [41]. It is worth stating that the production of secondary metabolites is strictly dependent on actinomycetes’ morphological and physiological cell differentiation [42]. Actinomycetes acquire resistance to their own antibiotics due to their resistant genes in order to prevent them committing suicide. However, under selective pressure such as the persistent exposure to antibiotics, these resistant genes are transferred, in a process called horizontal gene transfer, to other bacteria, including groups of pathogens [43,44].

Although actinomycetes are free-living organisms, some are opportunistic pathogens [38]. As well as their importance as a significant contributor to soil ecology in terms of degradation and the renewal of complex polymers such as chitin, cellulose, keratin and lignin [45], Actinobacteria are widely responsible for a growing number of antibiotic productions. It has been reported that around 500,000 natural compounds are derived from biological sources, and 70,000 of these natural compounds are of microbial origin, of which 29% are obtained from actinomycetes [46]. Actinomycetes are certainly the most lucrative and inexhaustible synthesizers of secondary metabolites with different ranges of biological activities among distinct microorganisms. Since the discovery of penicillin in 1928 by Alexander Fleming, about 80% of clinical antibiotics have come from the genus *Streptomyces* [47], with more than 500 of its species believed to be responsible for all of the bioactive metabolites discovered [48]. This genus is widely recognized for its extreme metabolic versatility. Actinobacteria are widely valued as a myriad resource in drug development with compounds that have cytotoxic/antitumor activity 39%, antimicrobial 31%, anti-HIV 1%, antimalarial 6%, antioxidant 2%, inhibitors 4%, antiinflammatory 5%, and other activity 10% [49]. Most of the active metabolites, including well-known drugs (erythromycin, streptomycin, tetracycline, antifungal compound nystatin, anthelmintic, avermectin, immunosuppressant rapamycin, and anticancer agents bleomycin and doxorubicin) have been shown to have distinctive structures and greater potency against infectious diseases [45]. The ability of actinomycete strains to produce a variety of antibiotics varies widely since some produce a single antibiotic, while others produce a wide range of antibiotics. With the development of high-end technologies, the genomes of actinomycetes are able to be sequenced, which has, relatively, unraveled their huge genomes and encoded a number of intriguing metabolites. Interestingly, each strain of actinomycete is genetically capable of producing 10–20 bioactive compounds [50]. Actinomycetes have a very differentiated and complex life cycle. They develop as mycelium and reproduce by sporulation [38]. Sometimes the growth or reproduction of actinomycetes is slow, so they are called slow growth. Most actinobacteria spend their life cycle as semi-dormant spores as a survival mechanism in the response to stress [38]. These spores are mostly resistant to desiccation, heating, some antibiotics and chemicals [46]. Under appropriate conditions, these spores are revived or reactivated, and their life cycle continues [38].

## 4. Marine Actinomycetes: A Potential Frontier of Bioactive Compounds

Marine actinomycetes are emerging as a promising candidate for bioactive metabolites, encompassing very complex compounds with pharmacological activities [51]. There is significant potential for the availability of bioactive compounds in marine actinomycetes, which are characterized as antimalarial, antibacterial, antifungal, anticancer, antitumor, antiinflammatory, cytotoxic and antimicrobial agents [52]. In addition to the synthesis of chemically active agents, marine actinomycetes promote the mineralization and degradation of organic matter and pollutants. They play a determining role in the biogeochemical processes of the oceans, thus maintaining the integrity of a particular marine environment [53]. Recent advances in marine drug research have mainly focused on marine actinomycetes, since two-thirds of polyketide drugs are obtained from this unique taxon [54]. Marine actinomycetes are valuable prokaryotes of economic and biotechnological importance. It has been reported previously that less than 1% of actinomycetes have been documented, in particular due to the dynamics and complexity of the microbial population [8]. Actinomycetes are widely distributed within ocean boundaries with highly developed morphological and cultural characteristics, so the majority remain untapped or elusive [22,55]. They are isolated from less extreme to extreme marine habitats, including sediment and seawater. Marine actinomycetes represent a tenth of all marine bacteria [39]. The extent of the diversity of marine actinomycetes is enormous due to the diversity of marine habitats. Several studies clearly documented the presence of marine actinobacteria by discovering *Rhodococcus marinonascene*, the first species of marine actinobacteria described [56]. However, it was widely believed that marine actinomycetes derived primarily from the dominant spores originated from the terrestrial ecosystem [57]. A number of studies have shown that strains of actinomycetes have developed certain characteristics of marine adaptation in order to survive [58], while others are metabolically active in the marine environment [59]. Compelling evidence regarding the widespread and persistent occurrence of native actinomycetes was first reported by Mincer et al. [60]. These findings subsequently paved the way for the discovery of a new genus of obligate marine actinobacteria *Salinispora* (also known as *Salinospora*) [61]. This new discovery clearly proves that actinomycetes are indigenous to the marine environment and are able to create unique bioactive compounds of interest to pharmaceutical researchers. Culture-independent studies have further proven that indigenous marine actinomycetes belong to the genera *Streptomyces*, *Dietzia*, *Solwaraspora*, *Williamsia*, *Marinispora*, *Verrucosispora*, *Aeromicrobium*, *Salinispora*, *Salinibacterium* and *Rhodococcus* [55,62].

An interesting recent review by Voser et al. [63] on “How different are marine microbial natural products compared to their terrestrial counterparts?” did an excellent job of summarizing 55,817 compounds reported from marine and terrestrial microorganisms and showed that 76.7% of the compounds isolated from marine microorganisms are closely related to compounds isolated from terrestrial microorganisms. They suggest that increasing incubation times and using specific culture-based methods that mimic marine environments is paramount for targeting unique marine actinobacteria compounds [63]. Having a holistic perspective on a variety of these diverse culture-based methods can help researchers design isolation methods accordingly, as the cost of marine research has been estimated to be an order of magnitude higher than equivalent land-based studies [64].

In this review, we support their conclusion and describe culture conditions and molecular biology protocols to help in their isolation and characterization.

## 5. Genomic Insight of Marine Actinomycetes

Metagenomic studies have given an insight into the evolutionary history, diversity and number of unculturable Actinobacteria, including those from marine habitats [65]. With the development and advancement of genome sequencing, an immense amount of DNA sequence data are available from public databases. The advent of the genomic era has revolutionized the approaches in drug discovery [66]. Genome mining is a powerful tool that has the ability to showcase the entire biosynthetic potential of a microbe [67,68]. For instance, genome mining approaches were employed to unveil the huge number of biosynthetic gene clusters for secondary metabolites from marine-derived actinomycetes. A study by Undabarrena et al. [69] not only showed that *Streptomyces* sp. H-KF8 isolated from marine sediments has 26 biosynthetic gene clusters for secondary metabolites, but also that it has the ability to tolerate a wide range of heavy metals. Another study unveiled 176 distinct biosynthetic gene clusters among three closely related species of the genus *Salinispora* isolated from various marine habitats of which 24 of the BGCs had a connection to their products [70]. Additionally, Xu et al. [71] analyzed 87 marine *Streptomyces* genomes isolated from marine sediments and invertebrates and revealed their number of secondary metabolite biosynthetic gene clusters, ranging from 16 to 84. Securing close and high-quality genomes remains vital to achieve accurate genome mining and in silico identification outputs of secondary metabolite biosynthetic gene clusters [72]. As more marine organisms are sequenced, the functions and applications of genome mining become more common in retrieving unique marine compounds [73]. However, genome mining for drug discovery from marine natural products is often challenged by predictions of chemical structures, novel classes and the activation of silent gene clusters [73]. The introduction of high throughput molecular techniques such as metagenomics is deemed appropriate for microbiota investigations that culture-based techniques have failed to investigate. With a richer knowledge and deeper insight into the functional characteristics of actinomycetes based on culture-independent studies, improved techniques have been developed to cultivate and recover previously uncultivable actinomycetes [74,75,76,77].

Indeed, the whole genome studies have revealed the immense potential of marine actinomycetes.

## 6. Cultivation Techniques of Marine Actinomycetes

Marine actinomycetes have special growing conditions that must be met for proper growth in the laboratory. A higher percentage of microbial cells in unexplored or underexplored habitats remain viable but uncultivable (VBNC) [72], as few microbial colonies can be isolated by conventional approaches in the laboratory. The isolation of actinomycetes from the marine environment requires expertise on the physiological characteristics of actinomycetes, and the taxonomies and isolation parameters such as the components and concentration of the medium, culture temperature, pH and incubation time [78]. Understanding these factors provides a higher success rate when it comes to isolating actinomycetes. To successfully grow marine actinomycetes under standard laboratory conditions, selective isolation approaches such as selective isolation media formulated with the preferred nutritional requirements of marine actinomycetes and the use of pretreatment to inhibit the growth of non-actinomycetes are essential. Studies have shown that nutrient-poor environments favor the isolation of rare marine actinomycetes over nutrient-rich environments [46]. Halophilic actinomycetes grow very slowly and, therefore, the isolation plates should have a lower substrate concentration and be thicker; consequently, they have a longer incubation period at appropriate temperatures [79]. In addition, the media must mimic the usual conditions of the microbe. With the abundance of sodium in the ocean, it is an essential prerequisite to add sodium chloride (NaCl) for the growth of most marine organisms. Thus, a well-designed growth medium should have osmotic values similar to those of seawater, which allows the efficient growth of marine actinomycetes [61,80]. This means that a growth medium should contain natural sea water, artificial sea water or deionized/distilled water with the addition of different concentrations of sodium chloride [46]. Moreover, different pretreatment methods should be used such as heat, physics, mechanics, the addition of chemicals or antibiotics, centrifugation, freezing, ultrasonic waves and radiation to remove or suppress the growth of non-actinomycetes (fast growing bacteria and fungi) [46,81]. To cultivate various actinomycetes, about three to five media with different ingredients and concentrations are used [82,83]. These media are able to restrict the growth of other microbes without harming the propagules of actinobacteria [78].

## 7. Fermentation and Extraction of Bioactive Compounds from Actinomycetes

Actinomycetes are known to produce pigments of orange, yellow, black or blue, greenish brown, pink, red and brown, depending on the type of strain grown, the isolation medium used and the age of the culture [38]. Actinomycetes also produce bioactive molecules in the form of secondary metabolites. The essence of the bioactive compounds generated by actinomycetes strongly depends on the species, strain and culture conditions. The production and secretion of antimicrobial agents do not have fixed yields but can be quantified or suppressed under different culture conditions [84,85]. Studies have shown that to produce the highest antimicrobial metabolites, the pH, incubation time, cell density, type and concentration of carbon sources (maltose, starch, glycerol and glucose) and nitrogen sources (ammonium chloride, soya bean meal, ammonium sulfate, yeast extract, peptone and ammonium nitrate) are essential [86]. Given the limited quantity of bioactive metabolites produced by various microorganisms, including actinomycetes, the applications of fermentation have received merited attention by pharmaceutical entities due to its feasibility for maximizing the production of the most commercial clinical drugs [84]. Fermentation is a biological process; it mostly involves microorganisms that regulate the enzymatic conversion of complex molecules into simple compounds [87]. In drug production, its pathway diverges conventionally into submerged/liquid fermentation (SmF) and solid-state fermentation (SSF) [88,89,90]. These foregoing versions of fermentation are mostly exploited due to their economic and environmental precedence. In spite of that, the outcome of each fermentation technique varies extensively in terms of substrate utilization and productivity [91]. Although SmF has gained wide recognition for its usage at a larger scale in terms of its bioactive secondary metabolites-producing capacity, SSF is emerging and advancing as a promising alternative to SmF [88]. At the research level, the active cultures of actinomycetes based on primary screening are generally cultivated in submerged culture or liquid media for secondary metabolites’ production with the added advantage of a feasible extraction and purification of natural compounds [92]. In order to recover bioactive compounds from the microbial fermentation culture, the extraction process is the first crucial step among a chain of techniques to segregate compounds of interest from raw materials. The extraction of natural compounds employs various extraction techniques, both conventional and modern, but mostly solvent-based extraction [93]. Conventional methods require an organic solvent or water, while modern techniques depend on an elevated temperature and pressure [94]. The Liquid–liquid extraction method or partitioning is commonly utilized for the extraction of secondary metabolites from actinobacteria [95]. Furthermore, the selection of the solvent is essential since extraction is based on the law of similarities and intermiscibility (polarities); both the solvent and solute should have near equivalent polarity values for efficient extraction [93]. The compounds present in the crude extract are rather complex with physical and chemical differences, thus requiring further separation and purification. Chromatography (thin-layer chromatography, column chromatography, and high-performance liquid chromatography) is the frequently used technique to acquire pure natural compounds [95].

## 8. The Biology of Coral

### 8.1. Coral Morphology and Distribution

Coral reefs are the main, unique and striking constituent of a marine ecosystem described as tropical underwater forests. They represent the most crucial bioconstruction on this planet and are described by both biological and geological entities [96]. Unlike tropical rainforests, coral reefs have higher biodiversity despite occupying only 0.1% of the Earth’s ocean surface. They are largely confined to tropical waters and play a central role in hosting a diverse marine flora and fauna, which is represented by more than 2 million species of marine life [97]. Coral reefs have an unusually complex structure, comprising various species of corals built by thousands of tiny, transparent nocturnal animals called coral polyps [98]. Corals are known for the simplicity of their body structure. A basic coral polyp has a sac-like body and mouth surrounded by a series of retractable tentacles that are concentrated with stinging cells called nematocysts. These stinging tentacles are used on purpose for self-defense or to capture food. Coral polyps have two distinct tissue layers: the gastrodermis and the epidermis, which are mostly separated by a simple gelatinous supporting matrix known as the mesoglea. These cell layers develop from the two germ layers, the endoderm and ectoderm, respectively, during the coral’s life cycle [99]. Corals are sessile invertebrates belonging to the class of Anthozoa within the phylum Cnidaria. They are present in a large numbers of species as well as in an abundance and live in colonies or solitaries and reproduce by sexual and asexual reproduction. Their sexual reproduction involves spawning and broadcast brooding, while asexual reproduction involves budding and fragmentation. There are two types of corals based on their physical texture, such as hard coral and soft coral. Hard corals, also known as scleractinian corals or stony corals, have an outer skeleton made of calcium carbonate (CaCO_3_) arranged in a crystalline form called aragonite. The stony corals that form the reefs are called hermatypic and they grow by shedding CaCO_3_ skeletons, a vital part of reef formation and structure. Hermatypic corals are the essential calcifying organisms that contribute to the biodiversity of coral reefs. On the other hand, soft or ahermatypic corals have small spikes of calcium carbonate embedded in their bodies. Unfortunately, they do not secrete CaCO_3_, so do not make a significant contribution to reef formation [98]. Since the ocean is the primary sink for carbon dioxide (CO_2_), it plays an important role in removing CO_2_ from the atmosphere, thereby regulating the earth’s climate and marine health [100]. The ocean, being an efficient CO_2_ absorber, allows trapped carbon dioxide molecules to be incorporated with calcium ions to form calcium carbonate in a process known as calcification via calcifying organisms. Corals are the main factory for calcium carbonate precipitates as well as other marine organisms, namely, molluscs, calcareous algae, foraminifera, sponges and echinoderms [101]. Corals depend on specific physiological and environmental requirements to support their survival. They live at temperatures ranging from 18 to 30 °C and have a salinity of 32 to 40% [102]. Corals are widely distributed in all of the world’s oceans but are limited between the latitudes of the Tropic of Cancer and the Tropic of Capricorn [98]. Their distribution is mainly determined by biotic factors (corallivores, coral’s intra and interspecific competitions; their reproductive and recovery capacity; and their ability to withstand environmental stress) and abiotic (light, water temperature, pH, salinity, turbidity and depth) [103]. Changing these parameters can have a dramatic impact on the health and survival of corals. Coral communities are found in shallow and deep ocean ecosystems.

### 8.2. Coral Significance and Bleaching

Coral reefs are among the most productive ecosystems that are ecologically and economically important to the livelihoods of millions of people and to marine life as a whole. The complex structures of coral reefs are an excellent habitat for marine organisms, as they provide a shelter, nursery and greater retention of nutrients as a food source for most marine species. The population as a whole depends on coral reefs: as a natural shoreline buffer against storm surges and wave erosion, and for protein needs, medicinal purposes and a lucrative source of income through the tourism and fishing industries [102,104,105]. Globally, the sustainability of corals is highly threatened by well-characterized phenomena, including natural and anthropogenic factors. Coral bleaching is a phenomenon that occurs due to a disruption of the symbiosis between corals and symbiotic algae. It is described as a loss of coral coloration when a coral polyp expels its zooxanthellae or there is a reduction in photosynthetic pigment in the zooxanthellae due to stressful triggers such as a variation in salinity, solar radiation, temperature, infection, cyclones, pollution and destructive human practices. Under these stressful conditions, certain functions of the coral are compromised, such as its capacity for growth and fecundity. This could then have lethal or sublethal consequences on the overall coral performance [106,107,108]. The recurrence and severity of these stressful triggers increases dramatically over time around the world. However, most corals have obtained a certain degree of tolerance against coral bleaching due to certain functional mechanisms. For instance, coral’s heat stress tolerance can be supported by heat shock proteins, the enhancement of antioxidant defense, photoprotective molecules (green fluorescent protein-like pigment and mycosporine-like amino acids) and host thermotolerant symbionts (uptake from the environment or reshuffle of the existing symbionts) [109].

### 8.3. Coral Defense Mechanism

Corals have a series of well-established and effective defence mechanisms that protect themselves from sedimentary organisms, sediments, pathogens and other potential threats. One of these defence mechanisms involves the production and release of mucus. The exudation of mucus films as a defence strategy coincides with the ability of corals to trap particles using secreted mucus. Coral mucus is synthesized by phlegm and its composition varies between coral species. Mucus is derived from photosynthesis products produced by symbiotic algae and compounds obtained via heterotrophic feeding. It is composed mainly of carbohydrates and mixtures of lipids, polysaccharides and glycoproteins (mucins). Several studies have characterized coral mucus as a nutrient-rich environment conducive to microbial growth and a potential source of energy for other marine organisms [110,111,112]. Mucus production has a number of protective measures. To begin with, beneficial mucus microbes act as a protective barrier against invading species via resource competition or the secretion of antimicrobial molecules [113,114]. In addition, mucus protects corals against solar radiation by generating proteins or pigments that absorb ultraviolet light [115]. Another relevant strategy is that when corals are exposed to sedimentation or air at low tide, mucus secretion prevents desiccation and suffocation [112]. Although corals are simple organisms, they have a complex immune system, which includes mechanisms capable of healing them from injuries and also the production of melanin as a defensive maneuver to get rid of or confine harmful bacteria [116]. Moreover, a major defence strategy used by corals is the digestion of phagocytic cells. Microscopic organisms are fought or degraded by enzymes and oxygen free radicals [117]. Defence levels vary among different families, genera and species of corals.

### 8.4. Coral Symbiosis

Corals harbor a large population of various microorganisms including bacteria, fungi, viruses and archaea as well as its symbiotic algae [118,119]. The coral symbionts offer significant contributions to the coral’s physiology, development, immunity and responses to fluctuating environmental conditions [120]. Recent culture-independent coral studies have shown that the microbial community inhabiting corals is highly diverse, abundant and rich in novel microbial species. Collectively, corals and their symbionts are characterized as a holobiont or coral microbiome. A coral microbiome is found in distinct parts of the coral, including the surface mucus layer, tissues and skeleton. Each coral compartment differs in its richness and microbial diversity [119,121,122]. For example, the microbial population of coral tissues is much more stable than that of surface mucus, which is constantly renewed. Environmental stressors can alter a coral’s microbiome, thereby compromising its immunity and allowing opportunistic microbes to thrive, which in turn drives coral motility [123,124]. A striking example of coral symbiosis includes the mutualistic relationship of corals with dinoflagellates zooxanthellae, symbiotic algal cells belonging to the genus *Symbiodinium* that inhabit their gastrodermis tissue, which enhances their growth and survival [125,126]. Corals have adapted to different mechanisms and strategies to take up *Symbiodinium* such as through their parents [127] or from adjacent seawater [128], where the dinoflagellates are constrained by a series of algal membranes embedded in an outermost membrane of coral origin; the entire membrane-bound organelle is called a symbiosome [129]. Vertical transmission of *Symbiodinium* species by the corals is correlated with a higher specificity in endosymbiosis union than horizontal acquisition [130].

Molecular analysis of the genus *Symbiodinium* has evidently revealed its diversity [125,131,132]. It is estimated that every square centimeter of the coral’s surface is filled with millions of these single-celled algae [125]. At different ocean depths, a single species of coral can be dominated by a single *Symbiodinium* type or can host many types of *Symbiodinium* in its anatomy [128]. There are certain physiological traits that differ among *Symbiodinium* types and their coral hosts, such as the thermal tolerance, growth rate, host infectivity, photophysiology and translocation of inorganic compounds [128,133].

*Symbiodinium* species can utilize light energy very efficiently [134]. These photosynthetic algae produce unique protein pigments that capture different wavelengths of light and emit colorful, vibrant colors displayed by diverse coral communities. In addition to this, some corals are biofluorescent under appropriate conditions due to fluorescent proteins [135]. There are corals that have zooxanthellae and others that do not. Corals without zooxanthellae exist in all the oceans of the world and depend entirely on zooplankton or particles apprehended by their tentacles for food. As these corals have no photosynthetic requirements, their growth is slower than those with zooxanthellae. Corals of this trait are able to live in shallow to deep water where there is no light [136]. In contrast, reef-building corals acquire their metabolic needs from microscopic organisms or particles and their symbiosis. Their dual dietary character is widely recognized as autotrophic and heterotrophic. Apparently, endosymbiotic dinoflagellates provide adequate nutrition in the form of organic molecules including glucose, fatty acids, glycerol and amino acids. In return, the corals provide protection, carbon dioxide, nitrogen and phosphorus to the symbiont for photosynthesis and cellular respiration [137,138]. A very vital element synthesized by symbiotic algae is the vast oxygen capacity provided to corals and its associated prokaryotes for efficient respiration [119]. The autotrophic supply of zooxanthellae is very crucial for the survival of corals, especially in nutrient-limiting ecosystems.

## 9. The Order Scleractinia

The order Scleractinia is classified in the subclass Hexacorallia within the class Anthozoa [139]. This order is comprised mainly of hard corals, which constitute the major architecture (formation and framework) of coral reefs. The Scleractinia constitute 31 families, about 240 genera and more than 1500 species. There are three categories of scleractinians, namely zooxanthellate species (corals that have symbiotic zooxanthellates), azooxanthellate species (those that do not have symbiotic zooxanthellates), and apozooxanthellate species (facultative zooxanthellate species). Most reef-building corals contain unicellular dinoflagellate zooxanthellae [140]. With the important contribution of corals to the major structure of calcium carbonate, scleractinians are considered hypercalcifiers; they are able to calcify quickly and massively thanks to the advantage of hosting the unicellular dinoflagellate [141]. The order Scleractinia has an ancient fossil lineage that dates back over 240 million years [142]. Their growth is strongly influenced by an increase in skeletal mass via calcification, tissue growth and zooxanthic growth. However, its growth is mainly determined by the accretion of carbonate deposits via calcification. The skeleton of a single coral polyp is called a corallite, with a replaceable basal plate that is constantly being created as the polyp grows. The skeletal structural deposits of individual polyps provide the basis for the colonization of larger numbers of coral polyps and other marine organisms. As corals proliferate, new skeletal materials are cobbled together over existing materials, resulting in the complexity of the calcium carbonate edifice. Upward growth from a CaCO_3_ platform allows new polyps to bud from existing polyps or grow between two existing polyps. The majority of Scleractinia are colonial modular organisms, interconnected by a series of polyps that share continuous tissue. Hermatypic corals prefer shallow, clear, warm tropical or subtropical waters to grow and thrive. In these oligotrophic circumstances, there is adequate light energy and constant wave action, which pulls the corals away from sediments that could hinder or reduce their effectiveness. However, at extreme depths these corals grow outward at an angle to avoid being overwhelmed by sediment and to catch reducing light [98]. Hard corals have different shapes or growth forms including mushroom, tuft, branch, table, elkhorn, digitate, cup/flower, sub-clump and encrusted. Each growth form is primarily species-specific; it is therefore correlated with the growth rate of corals. Coral growth forms are adapted to a certain location. For example, corals located in a place where the waves are constant have mound shapes, while those that are not have complex shapes such as branching [98]. Scleractinian corals have employed a very sophisticated defense mechanism in response to invasion by adjacent benthic organisms. These mechanisms involve an exponential growth rate, coral necrosis via digestive enzymes secreted by the mesenteric filaments outwards and allelochemical secretions [143].

## 10. Association between Scleractinian and Actinomycetes

Actinomycetes residing in marine habitats, and especially those obtained from marine organisms, offer a diverse myriad of bioactive substances, which are stimulated by the ecological interactions of the hosts. Several of these marine sources, including sponges, marine sediments, crabs, ascidians, marine substrates, sea cucumbers, corals, fish and algae, have a symbiotic relationship with actinomycetes [49]. These taxa have come under scrutiny for being specialized metabolites. Marine sponges generate key structures that are used as therapeutic agents and therefore represent a remarkable source of bioactive metabolites [144]. They have long been recognized as an intriguing group for drug discovery [145]. Nevertheless, corals are ranked as the second most productive source of natural products extracted from marine organisms after sponges [146]. To date, 5800 compounds have been derived from corals, contributing nearly 20% of all total marine natural products [147]. Marine microbes often synthesize minute amounts of bioactive metabolites. Although marine organisms can produce various bioactive compounds, their pharmaceutical potentials are determined by a supply problem [148,149]. For the production of medicines, collecting a large quantity of marine organisms is not a sustainable approach. Thus, studies have gradually shifted towards the host of associated microbes since microorganisms also offer various valuable bioactive compounds [150]. In the last blackout (2010–2019), many studies described natural coral products in relation to coral-derived microorganisms. However, during the same period, the number of publications focusing on new compounds in relation to microorganisms associated with corals increased slightly, in contrast to those targeting only corals [151]. Actinobacteria are key organisms that regulate coral health. Among other functions of actinobacteria associated with corals, it is suggested that potential roles of actinobacteria in corals involve nutrient supply and recycling for protection against pathogens. They could have a high specificity and could be an omnipresent symbiont of corals [152,153]. Their microbial composition or structure changes when corals are exposed to stresses. For example, Cárdenas et al. [154] reported six species of actinobacteria from diseased white plague corals and a single species from healthy corals. An increase in algal cover or high temperatures usually coincide with a decrease in the population of actinobacteria in corals [155]. This further suggests the beneficial role of actinobacteria in the coral microbiome and as a bio-indicator of environmental stress due to their higher sensitivity. The first evidence of actinomycetes recovered from corals was presented by a culture-dependent study [156]. Phylogenetic analyses have further proven that the abundance of actinomycetes in corals differs among coral species and varies from 10 to 50% of the total coral bacteria [157]. Another study reported for the first time the isolation of actinobacteria from both mucus and coral tissue with antibacterial activities [158]. To date, very little information is available on the diversity of actinomycetes associated with corals. Yang et al. [159] reported the different cultivable actinobacteria of soft and hard corals. Actinobacteria are normally isolated from coral tissue and mucus with differences in abundance and diversity [23]. However, there are not enough studies that focus on isolating actinobacteria and their produced metabolites from scleractinian corals [160]. Corals produce a variety of specialized metabolites, but mounting evidence shows that the majority of these bioactive compounds are generated by symbiotic microbes [161]. Due to this growing recognition, more attention is being paid to the associated microbiota. Nevertheless, the discovery of natural products is uneven between soft corals and hard corals, where only 3% of secondary metabolites discovered come from hard corals. Interestingly, hard corals have a higher percentage of symbiotic bacterial isolates than soft corals [162].

The coral-derived actinobacteria are capable of producing bioactive compounds (Table 1). A study isolated actinomycete strains belonging to genera *Streptomyces*, *Microbacterium*, *Cellulosimicrobium*, *Nocardiopsis*, *Mycobacterium*, *Micromonospora*, *Brevibacterium*, *Tsukamurella*, *Gordonia*, *Micrococcus*, *Jiangella*, *Brachybacterium*, *Prauserella*, *Pseudonocardia* and *Amycolatopsis* from scleractinian corals. The data unfold the genetic capacity of actinomycetes associated with stony corals that are capable of producing potent secondary metabolites [163]. Another study isolated actinobacteria relating to the genera *Streptomyces*, *Micrococcus*, *Curtobacterium* and *Propionibacterium* from the coral *Acropora digitifera* mucus. The actinomycete strains were analysed for their antimicrobial activity against various human pathogens such as *Staphylococcus aureus* and showed effective antimicrobial activity [160]. It was reported that only medicinal plants were able to inhibit the biofilm formation of *Streptococcus pyogenes* [164]. However, the isolated actinomycete species, especially *Streptomyces akiyoshinensis* and *Actinobacterium* sp., displayed efficient potency with extracts showing 60–80% of antibiofilm activity [165]. Approximately 23% of actinobacteria isolates obtained from *Acropora clathrata* and *Porites compressa* mucus are able to degrade most of the petroleum and oil toxicity [166]. The species *Rothia amarae* is capable of producing a signaling compound known as autoinductor 2, which is used in quorum sensing when exposed to stress events [167]. Some actinobacteria (*Brevibacterium* sp., *Kytococcus* sp., *and Brachybacterium* sp.) isolated from corals are regarded as organophosphate pesticide degraders, this activity protects corals from pesticide contamination [168]. Additionally, deep sea corals are emerging as a potential research area due to their abilities to have antifungal, antibacterial and cytotoxic activities [169,170]. New actinobacteria are being isolated from corals as studies multiply in this habitat. To illustrate, studies have unveiled the genera of actinobacteria *Dermacoccus* and *Serinicoccus* [159] and the actinobacteria strains *Brachybacterium paraconglomeratum* and *Brevibacterium linesis* [158] from the stony corals for the first time. They are classified under different actinobacteria families such as Dermacoccaceae, Ornithinimicrobiaceae, Dermabacteriaceae and Brevibacteriaceae, respectively (Table 1). Moreover, additional studies have further isolated actinobacteria from stony corals that have antimicrobial activity [23,171], antifungal activity [172,173], antibacterial activity [158,174,175,176], plant biostimulant activity [177] and potent biosurfactant anticancer activity [178], and those that have had no bioactivity test performed [154,156,179,180,181,182] (Table 1).

## 11. Novel Species and Compounds of Actinobacteria Recovered from Coral (Order Scleractinia)

**Figure 1:**
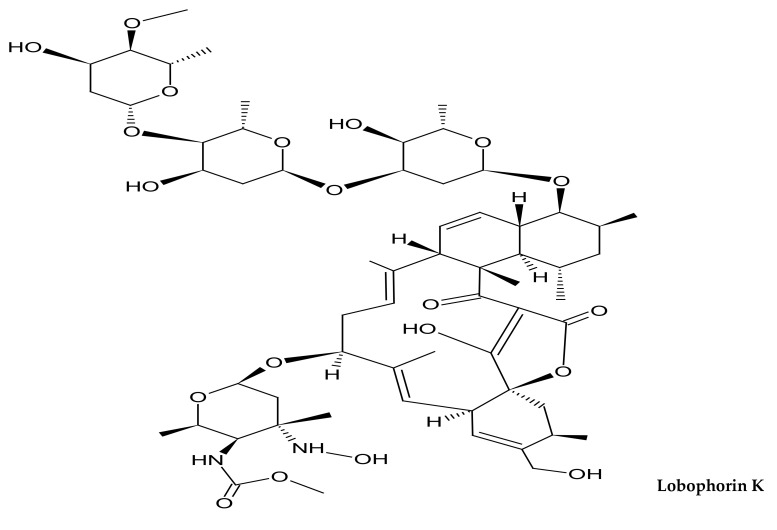

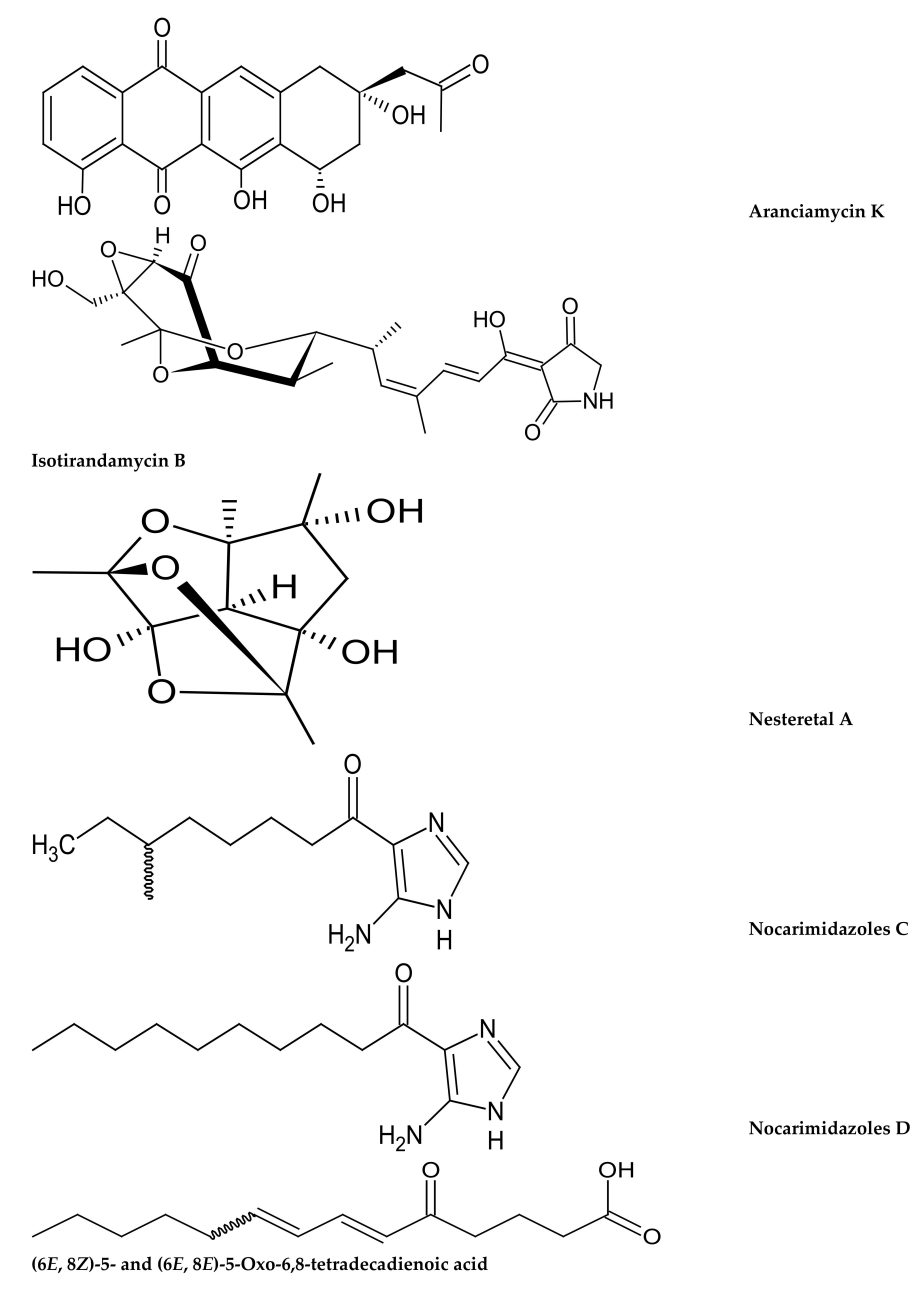

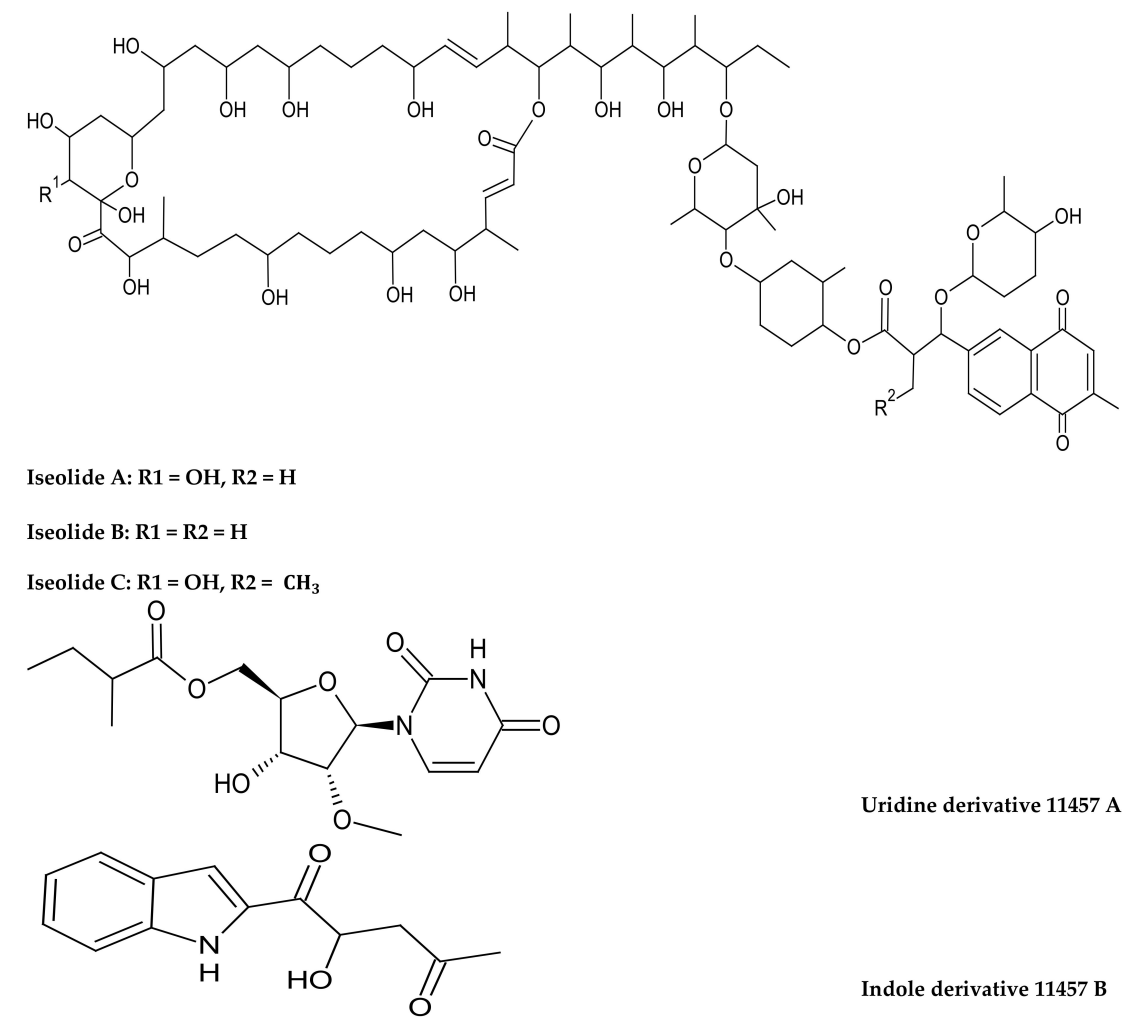
Structures of newly reported compounds of marine actinomycetes recovered from scleractinian corals between 2007 and 2022.

A total of 11 new actinomycete species belonging to nine different actinomycete families were recovered from stony corals between 2007 and 2022 (Table 2). The families of actinomycetes reported in hard corals to which the species belong are Intrasporangiaceae (1 species) [183], Pseudonocardiaceae (2 species) [184,185], Nocardiaceae (1 species) [186], Nocardiopsaceae (1 species) [187], Kineosporiaceae (2 species) [188,189], Promicromonosporaceae (1 species) [190], Corynebacteriaceae (1 species) [191], Micrococcaceae (1 species) [192] and Streptomycetaceae (1 species) [189]. The amount of new actinomycete species discovered is limited despite a review period of more than a decade. Regarding new compounds, a total of 13 different new compounds were reported for five genera of actinomycetes from 2017 to 2022 (Table 3). Prior to this given period, no new actinomycete compounds were reported in scleractinian corals. This is supported by Raina et al. [193], whose study was the first to isolate and identify an antimicrobial compound produced by bacteria associated with reef-building coral. The new compounds identified in this review belong to the genera *Kocuria* (2 new compounds) [194], *Pseudonocardia* (2 new compounds) [195], *Micrococcus* (2 new compounds) [176], *Streptomyces* (6 new compounds) [173,196,197] and *Nesterenkonia* (1 new compound) [198]. Among them, only the new compounds isolated from the genus *Pseudonocardia* show no bioactivity, while other genera have compounds with different degrees of antimicrobial characteristics. In comparison to other actinomycete genera, the genus *Streptomyces* has the highest number of new compounds discovered from hard corals. The structures of the reported novel compounds are highlighted in Figure 1. These compounds belong to different chemical classes such as alkanoilimidazoles, alkaloids, keto fatty acids, macrolides, spirotetronate, anthracycline, tirandamycin and polyketide. As shown in Table 2 and Table 3, more new species and compounds of actinomycetes have been discovered as of the year 2020.

## 12. Conclusions

A wide range of actinomycetes have been isolated from scleractinian corals with different bioactivities. The use of a growth medium with natural seawater or artificial seawater and distilled water diluted with different concentrations of sodium chloride as well as antifungal and antibacterial agents promotes the culture of marine actinomycetes from scleractinian corals. Most available studies largely focus on the isolation of actinomycetes from coral mucus, followed by its tissue and skeleton. This review presents 11 new species of actinobacteria as well as 13 new compounds obtained from actinomycetes in corals. In addition, it summarizes recent knowledge on the diversity, isolation and distribution of actinomycetes in scleractinian corals. The study of the functions of actinobacteria in the coral as well as the exploration of their capacity for biotechnological purposes is an area of growing interest in the scientific community. Thus, the coral-derived actinomycete and its specialized metabolites isolated so far represent only a small part of the immense diversity of marine actinomycetes. For future reference, more research should target coral actinomycetes with improved selective isolation approaches and techniques. Pacific island countries are home to a vast pristine coral environment and represent an unlimited source of potential new bioactive compounds from actinobacteria.

## Figures and Tables

**Table 1:** Genus/species of Actinobacteria isolated from coral (order Scleractinia) between 2006 and 2022.

Genus/Species	Family	Coral Species and Nature of Sample	Isolation Medium, Incubation Temperature/Time, Pretreatment and Bioactivity	Country/Sampling Location	Ref.
*Micrococcus luteus* *Dermatophilus congolensis* *Kytococcus sedentarius* *Kocuria* sp.	Micrococcaceae Dermatophilaceae Kytococcaceae	*Fungia scutaria* (depth of 1–2 m) (mucus)	Bacto Marine Agar 2216 + salt concentration (3.6%). Incubated at 25 °C for 3–7 days. (No bioactivity test)	Gulf of Eilat, northern Red Sea.	[156]
*Actinomyces* sp. *Micrococcus roseus* *Micrococcus* sp. *Micrococcus varians*	Actinomycetaceae Micrococcaceae	*Acropora cervicornis* (depth of 4–5 m) (mucus)	Sucrose seawater medium. Incubated at 28 °C for 24 h. (No bioactivity test)	North of Bidong Island, Terengganu, Malaysia	[182]
*Brachybacterium* sp. *Brevibacterium* sp. *Kytococcus* sp.	Dermabacteriaceae Brevibacteriaceae Kytococcaceae	*Porites* sp. *Galaxea* sp. *Acopora* sp. *Pavona* sp. (depth of 2–3 m) (tissue)	Half-strength Zobell 2216E marine agar. Incubated at room temperature for 48 h. (Capable of degrading organophosphorous pesticides)	Java, Bali, Sulawesi and Komodo Island	[168]
*Brachybacterium paraconglomeratum* *Brevibacterium linesis* *Kocuria rosea* *Kocuria flavus*	Dermabacteriaceae Brevibacteriaceae Micrococcaceae	*Acropora digitifera* (mucus and tissue slurry)	Zobell Marine agar. Incubated at 27 °C for 7–12 days. (Antibacterial activity)	India (gulf of manner)	[158]
*Streptomyces akiyoshinensis* *Actinobacterium* sp.	Streptomycetaceae Actinomycetaceae	*Acropora digitifera* (mucus)	Starch casein agar. Incubated at 27 °C for 7–12 days. (Antibiofilm activity)	India (gulf of manner)	[165]
*Streptomyces* *Micrococcus* *Curtobacterium* *Propionibacterium*	Streptomycetaceae Micrococcaceae Microbacteriaceae Propionibacteriaceae	*Acropora digitifera* (mucus)	Starch casein agar + actidione (40 µgml^−1^) and nalidixic acid (10 µgml^−1^). Incubated at 28 °C for 10–14 days (Antibacterial activity)	India (gulf of manner)	[160]
*Micrococcus luteus* *Mycrobacterium paraoxydans* *Brachybacterium paraconglomeratum* *Kocuria rhizophila* *Kytococcus sedentarius*	Micrococcaceae Microbacteriaceae Dermabacteraceae Kytococcaceae	*Acropora hyacinthus* (depth < 10 m) (mucus)	Marine agar. Incubated at 28 °C for 48 h. (No bioactivity test)	Vatia Bay, American Samoa	[181]
*Nocardiopsis alba* *Micrococcus yunnanensis*	Nocardiopsaceae Micrococcaceae	*Diploria strigosa* (depth of 15 m) (crushed coral)	Luria Bertani agar and Marine agar. Incubated at 28 °C for 48 h. (No bioactivity test)	Aguja Island, Panama	[154]
*Brevibacterium linens* *Leucobacter komagatae* *Microbacterium arabinogalactanolyticum* *Microbacterium oxydans* *Brevibacterium fermense* *Dietzia* sp.	Brevibacteriaceae Microbacteriaceae Dietziaceae	*Siderastrea siderea* (depth of 15 m) (crushed coral)			
*Rothia amarae*	Micrococcaceae	*Platygra carnosus* (crushed coral)	Heterotrophic bacterial media + artificial seawater. Incubated at 20 °C for 48–72 h. (No bioactivity test)	Hoi Marine Park, China	[180]
*Dietzia maris* *Gordonia bronchialis* *Gordonia lacunae* *Kocuria flava* *Micrococcus luteus* *Mycobacterium* *Chlorophenolicum*	Dietziaceae Gordoniaceae Micrococcaceae Microbacteriaceae	*Acropora clathrata* *Porites compressa* (mucus and tissue)	Basal Inorganic Medium + 0.5% light crude oil (carbon source). Incubated at 30 °C for 1 week. (Oil degraders)	Kuwait	[166]
*Dermacoccus* sp. *Serinicoccus* sp. *Rhodococcus* sp. *Nacardioides* sp. *Micromonospora* sp. *Leucobacter* sp. *Brevibacterium* sp.	Dermacoccaceae Ornithinimicrobiaceae Nocardiaceae Nocardioidaceae Micromonosporaceae Microbacteriaceae Brevibacteriaceae	*Tubastraea coccinea* (depth of 5–10 m) (crushed coral)	Glycerol arginine agar (GAA), asparagine peptone agar (APA) and R2A + ASW + potassium dichromate (50 μgml^−1^) with nalidixic acid (15 μgml^−1^). Incubated at 28 °C for 3–6 weeks. (No bioactivity test)	Zhao’an Bay, East China Sea	[159]
*Streptomyces variabilis* *Streptomyces rutgersensis* *Streptomyces viridodiastaticus* *Mycobacterium gilvum* *Mycobacterium parafortuitum* *Mycobacterium vanbaalenii* *Nocardiopsis yanglingensis* *Micromonospora aurantiaca* *Brevibacterium epidermidis* *Brevibacterium picturae* *Gordonia* sp. *Gordonia westfalica* *Brachybacterium paraconglomeratum* *Cellulosimicrobium funkei*	Streptomycetaceae Mycobacteriaceae Nocardiopsaceae Micromonosporaceae Brevibacteriaceae Gordoniaceae Dermabacteraceae Promicromonosporaceae	*Porites lutea* (depth of 3–5 m) (tissue)	Marine agar, yeast extract agar, trehalose proline agar, raffinose histidine agar and pyruvic acid sodium asparagine agar + natural seawater. Media pH-7.5, Incubated at 28 °C for 3–4 weeks. (Antimicrobial activity)	South China sea	[163]
*Streptomyces variabilis* *Streptomyces fimicarius* *Micrococcus yunnanensis* *Microbacterium aerolatum* *Nocardiopsis flavescens* *Nocardiopsis dassonvillei* *Nocardiopsis alba* *Micromonospora aurantiaca Brevibacterium epidermidis* *Brevibacterium picturae* *Tsukamurella pulmonis* *Gordonia* sp. *Amycolatopsis* sp. *Jiangella alba* *Jiangella gansuensis* *Brachybacterium paraconglomeratum* *Prauserella marina* *Pseudonocardia kongjuensis*	Streptomycetaceae Micrococcaceae Microbacteriaceae Nocardiopsaceae Micromonosporaceae Brevibacteriaceae Tsukamurellaceae Gordoniaceae Pseudonocardiaceae Jiangellaceae Dermabacteraceae	*Galaxea fascicularies* (depth of 3–5 m) (tissue)			
*Streptomyces fimicarius* *Microbacterium ginsengisoli* *Microbacterium paraoxidans* *Nocardiopsis flavescens* *Nocardiopsis yanglingensis* *Micromonospora aurantiaca Brevibacterium epidermidis* *Gordonia* sp. *Jiangella gansuensis* *Pseudonocardia carboxydivorans* *Pseudonocardia ammoniooxydans* *Cellulosmicrobium funkei*	Streptomycetaceae Microbacteriaceae Nocardiopsaceae Micromonosporaceae Brevibacteriaceae Gordoniaceae Jiangellaceae Pseudonocardiaceae Promicromonosporaceae	*Acropora millepora* (depth of 3–5 m) (tissue)			
*Streptomyces cyaneofuscatus* *Streptomyces carnosus*	Streptomycetaceae	Deep sea corals; *Lophelia pertusa*, and *Desmophyllum* sp. (depth of 1800 m) (crushed coral)	One-third tryptic soy agar (TSA) and 1/6 MOPS BLEB agar (Oxoid) + seawater + cycloheximide (80 µgml^−1^) and nalidixic acid (20 µgm^−1^). Incubated at 28 °C for 2 weeks. (antibacterial, antifungal, cytotoxic, antiinflammatory activities)	Aviles Canyon, Cantabrian sea.	[169]
*Streptomyces* sp. *Rhodococcus* sp. *Nocardia* sp. *Brevibacterium* sp. *Micrococcus* sp. *Devriesea* sp. *Kocuria* sp. *Cellulomonas* sp. *Arthrobacter* sp. *Dermacoccus* sp.	Streptomycetaceae Nocardiaceae Brevibacteriaceae Micrococcaceae Dermabacteraceae Cellulomonadaceae Dermacoccaceae	*Coscinaraea columna* (mucus, tissue and skeleton)	R2A medium, M2 medium, M4 medium and Starch Casein Agar (SCA). R2A and SCA media + 3% (*w*/*v*) NaCl + potassium dichromate (50 µgml^−1^), nalidixic acid (15 µg ml^−1^), cycloheximide (75 µgml^−1^) and nystatin (75 µgml^−1^). Media pH-7.6, incubated at 28–30 °C for 3–6 weeks. (Antimicrobial activity)	Kuwait	[23]
*Streptomyces* sp. *Rhodococcus* sp. *Micromonospora* sp. *Dietzia* sp. *Brevibacterium* sp. *Micrococcus* sp. *Brachybacterium* sp. *Kocuria* sp. *Microbacterium* sp. *Cellulomonas* sp. *Arthrobacter* sp. *Ornithinimicrobium* sp. *Kineococcus* sp. *Agrococcus* sp.	Streptomycetaceae Nocardiaceae Micromonosporaceae Dietziaceae Brevibacteriaceae Micrococcaceae Dermabacteraceae Microbacteriaceae Cellulomonadaceae Ornithinimicrobiaceae Kineosporiaceae	*Platygyra daedalea* (mucus, tissue and skeleton)			
*Streptomyces* sp. *Rhodococcus* sp. *Nocardia* sp. *Brevibacterium* sp. *Micrococcus* sp. *Kocuria* sp. *Microbacterium* sp. *Arthrobacter* sp. *Marmoricola* sp.	Streptomycetaceae Nocardiaceae Brevibacteriaceae Micrococcaceae Microbacteriaceae Nocardioidaceae	*Porites harrisoni* (mucus, tissue and tissue)			
Not specified; Actinomycete isolates with different codes including a prominent antifungal code SCAS324		*Goniopora* spp. and *Porites* spp. (mucus)	Starch nitrate agar and starch casein agar + seawater. Media pH-7.2 and 7.4, respectively. Incubated at 30 °C for 4 weeks. (Antifungal activity)	Indonesia	[172]
*Rothia amarae*	Micrococcaceae	*Galaxea* sp. *Porites lutea* (mucus)	Marine agar 2216. Incubated at 30 °C for 1–2 weeks. (Produces autoinductor 2)	China	[167]
*Streptomyces* sp.	Streptomycetaceae	Deep sea corals (depth of 2000 m) (crushed coral)	One-third tryptic soy agar (TSA) and 1/6 MOPS BLEB agar (Oxoid) + seawater + cycloheximide (80 µgml^−1^) and nalidixic acid (20 µgml^−1^). Incubated at 28 °C for 2 weeks. (Antifungal and antibacterial activities)	Aviles Canyon, Cantabrian sea.	[170]
*Kocuria turfanensis*	Micrococcaceae	*Porites panamensis* (depth of 2–10 m) (crushed coral)	Marine agar. (No bioactivity test)	Gulf of California, Mexico	[179]
*Micromonospora marina*	Micromonosporaceae	*Acropora formosa* (mucus)	Starch casein agar + cycloheximide and nalidixic acid. Incubated for 7 days. Pre-heat treatment before serial dilution. (Potent biosurfactant-anticancer)	Not stated	[178]
*Streptomyces* sp.	Streptomycetaceae	*Dendrophyllia* sp. (depth of 20 to 25 m) (crushed coral)	ISP4 agar medium. Incubated at 23 °C for 14 days. (Antifungal activity)	Japan	[173]
*Micrococcus* sp.	Micrococcaceae	*Catalaphyllia* sp. (crushed coral)	Marine agar 2216 (Difco). Incubated at 23 °C for 2 days. (Antibacterial activity)	Japan	[176]
*Salinispora arenicola*	Micromonosporaceae	*Porites lobata* *Porites panamensis* (tissue)	Ten percent A1 culture medium + cycloheximide (100 μgml^−1^) and gentamicin (5 μgml^−1^). Incubated at 28 °C for 2 weeks. Coral tissue dried in laminar flow hood for 72 h before serial dilution. (Plant biostimulant activity)	Tropical central Pacific	[177]
*Streptomyces* sp.	Streptomycetaceae	Unidentified stony corals (crushed coral)	Nine different enrichment media; M1 (Mycose agar), M2 (Actinomycete isolation agar), M3 (Glucose asparagine agar), M4 (International Streptomyces Project), M5 (Humic Vitamin acid agar), M6 (Glycerin agar), M7 (Chitin agar), M8 (Gauze’s no.1 agar) and M9 (Marine agar) + nystatin (50 µgml^−1^) and trimethoprim (50 µgml^−1^). Incubated at room temperature for 3–5 weeks. Wet heat treatment (55 °C for 5 min) and incubated at 28 °C in a rotary shaker at 200 rpm for 30 min to facilitate the growth of actinomycetes. (Antibacterial activity)	South China Sea	[174]
*Streptomyces* sp.	Streptomycetaceae	Unidentified hard coral sample (crushed coral)	ISP2 medium + 0–5% NaCl + nystatin (100 µgml^−1^) and nalidixic (100 µgml^−1^). Incubated at 27–32 °C (Antibacterial activity)	Indonesia	[175]
*Glutamicibacter mysorens*	Micrococcaceae	*Favites halicora* (mucus)	Nutrient agar, marine agar, R2A agar, starch casein agar and International Streptomyces Project medium-2 (ISP-2) agar + seawater + cycloheximide (100 μgml^−1^) and nalidixic acid (25 μgml^−1^). Incubated at 26 ± 2 °C for 3 weeks. (Antimicrobial activity)	Southeast coast of India	[171]

**Table 2:** New species of Actinobacteria from coral (order Scleractinia) reported between 2007 to 2022.

Novel Species	Family	Coral Species and Nature of Sample	Isolation Medium and Incubation Temperature/Time	Ref.
*Janibacter corallicola*	Intrasporangiaceae	*Acropora gemmifera* (depth of 3–5 m) (crushed coral)	One-tenth MA + Ca medium. Incubated at 25 °C for about 1–3 weeks.	[183]
*Corynebacterium maris*	Corynebacteriaceae	*Fungia granulosa* (depth of 10–15 m) (coral mucus)	Marine agar, LB agar and nutrient agar. Incubated at 30 °C for 48 to 72 h.	[191]
*Prauserella coralliicola*	Pseudonocardiaceae	*Galaxea fascicularis* (depth of 5 m) (tissue slurry)	Isolation medium (yeast extract 0.25 g, $K_{2}{\mathrm{HPO}}_{4}$ 0.5 g, agar 12 g, 500 mL seawater and 500 mL distilled water; pH 7.0). Incubated at 28 °C for 4 weeks.	[184]
*Myceligenerans cantabricum*	Promicromonosporaceae	Deep sea coral (order: Scleractinia, Family: *Caryophillidae*) (depth of 1500 m) (crushed coral)	Selective media; 1/3 tryptic soy (TSA, Merck) and 1/6 M-BLEB agar + seawater + cycloheximide (80 µgml^−1^) and nalidixic acid (20 µgml^−1^).	[190]
*Rhodococcus electrodiphilus*	Nocardiaceae	An unidentified stony coral sample (crushed coral)	Marine agar (MA; Hi-Media) incubated at 25 °C for 1 week.	[186]
*Saccharopolyspora coralli.*	Pseudonocardiaceae	*Porites* sp. (depth of 5 m) (crushed coral)	ISP media 2 & 4 (fast growth), 3 & 6 (slow growth) and TSA (fast growth). Incubated at 25–30 °C for 3 weeks.	[185]
*Pseudokineococcus galaxeicola*	Kineosporiaceae	*Galaxea* sp. (depth of 4.2 m) (coral mucus)	Mucus agar medium. Cultivated at 25 °C for 30 days.	[188]
*Glutamicibacter mishrai*	Micrococcaceae	*Favia veroni*	Isolation medium; Marine agar 2216. Incubated at 25 °C for 4 days.	[192]
*Nocardiopsis coralli*	Nocardiopsaceae	*Galaxea astreata* (crushed coral)	Gause modified medium 1 + potassium dichromate (75 µgml^−1^). Cultivated at 28 °C for 4 weeks.	[187]
*Streptomyces corallincola* *Kineosporia corallincola*	Streptomycetaceae Kineosporiaceae	*Favites pentagona*		[189]

**Table 3:** New compounds of Actinobacteria from corals (order Scleractinia) reported between 2017 to 2022.

New Compound	Chemical Class	Source	Bioactivity	Ref.
Lobophorin K	Spirotetronate	*Streptomyces* sp. from deep sea coral *Lophelia pertusa*	Cytotoxic activity	[196]
Aranciamycin K Isotirandamycin B	Anthracycline Tirandamycin	*Streptomyces* sp. from *Porites* sp.	Isotirandamycin B showed antimicrobial activity against *Streptococcus agalactiae* with a MIC of 11.5 μM	[197]
Nesteretal A	Polyketide	*Nesterenkonia halobia* from scleratinian coral *Platygyra*	Showed a weak retinoid X receptor-α transcriptional activation effect	[198]
Nocarimidazoles C Nocarimidazoles D	Alkanoylimidazoles	*Kocuria* sp from stony coral *mycedium* sp.	Moderate antimicrobial activity against gram-positive bacteria and fungi (MIC—6.25–25 μg/mL)	[194]
(6*E*, 8*Z*)-5- and (6*E*, 8*E*)-5- Oxo-6,8-tetradecadienoic acid	Keto fatty acids	Actinomycete from the genera *Micrococcus* sp. which is associated with stony coral *Catalaphyllia* sp.	Showed antibacterial activity against the plant pathogen *Rhizobium radiobacter* and the fish pathogen *Tenacibaculum maritimum*.	[176]
Iseolide A Iseolide B Iseolide C	Macrolides	*Streptomyces* sp. associated with the stony coral of the genus *Dendrophyllia*	Showed antifungal activity against the plant pathogen *Glomerella cingulate* and human pathogens *Candida albicans* and *Trichophyton rubrum*. (MIC—6.25–25 μg/mL)	[173]
Uridine derivative 11457 A Indole derivative 11457 B	Alkaloids	*Pseudonocardia* sp. from the stony coral *Galaxea fascicularis*	Showed no antibacterial activity against pathogenic bacteria and cytotoxic against human cancer cell lines	[195]

## Data Availability

Not applicable.
